# Metagenomic Detection of Multiple Viruses in Monk Parakeet (*Myiopsitta monachus*) in Australia

**DOI:** 10.1002/vms3.70083

**Published:** 2024-10-18

**Authors:** Babu Kanti Nath, Suman Das Gupta, Saranika talukder, Nasrin Sultana Tonu, Shane R. Raidal, Jade K. Forwood, Subir Sarker

**Affiliations:** ^1^ School of Agricultural Environmental and Veterinary Sciences Faculty of Science and Health Charles Sturt University Wagga Wagga New South Wales Australia; ^2^ Biosecurity Research Program and Training Centre Gulbali Institute Charles Sturt University Wagga Wagga New South Wales Australia; ^3^ College of Public Health Medical and Veterinary Sciences James Cook University Townsville Queensland Australia; ^4^ Upazila Livestock Office and Veterinary Hospital Cumilla Bangladesh; ^5^ School of Dentistry and Medical Sciences Faculty of Science and Health Charles Sturt University Wagga Wagga New South Wales Australia; ^6^ Biomedical Sciences & Molecular Biology College of Public Health Medical and Veterinary Sciences James Cook University Townsville Queensland Australia; ^7^ Australian Institute of Tropical Health and Medicine James Cook University Townsville Queensland Australia; ^8^ Department of Microbiology Anatomy, Physiology, and Pharmacology School of Agriculture Biomedicine and Environment La Trobe University Melbourne Victoria Australia

**Keywords:** adenovirus, beak and feather disease virus, chaphamaparvovirus, metagenomics, next‐generation sequencing, parrot

## Abstract

**Background:**

Birds are known to harbour many pathogens, including circovirus, herpesviruses, adenoviruses and *Chlamydia psittaci*. Some of these pose zoonotic risks, while others, such as beak and feather disease virus (BFDV), have a significant impact on the conservation of endangered bird species.

**Objectives:**

This study was aimed to determine the faecal virome of a group of apparently healthy Monk parakeet using high‐throughput sequencing.

**Methods:**

Fresh faecal samples were collected from four Monk parakeets at a pet shop in Melbourne, Australia. Virus enrichment and nucleic acid extraction were performed on the faecal samples, followed by high‐throughput sequencing at the Australian Genome Research Facility (AGRF).

**Results:**

Utilising an established pipeline for high‐throughput sequencing data analysis, this study revealed the presence of three viruses of the families *Circoviridae*, *Parvoviridae* and *Adenoviridae*. Subsequent sequence comparison and phylogenetic analyses further confirmed that the detected viruses belong to the genera *Chaphamaparvovirus* (unassigned species), *Circovirus* (species *Circovirus parrot*) and *Siadenovirus* (species *Siadenovirus viridis*).

**Conclusion:**

Despite non‐pathogenicity, the existence of multiple viruses within a bird species underscores the risk of these viruses spreading into the pet trade. Detection and a better understanding of avian viruses are crucial for the establishment of appropriate management and biosecurity measures in the domestic and international bird trade, which ultimately supports the conservation of vulnerable bird species.

## Graphical abstract

Summary

Three viruses from the families *Circoviridae*, *Parvoviridae* and *Adenoviridae* were detected in this study using a non‐invasive, high‐throughput sequencing method.

Though their pathogenic consequences are unknown, these viruses may pose a risk of spreading infection within the pet trade.

This study also highlights the importance of a deeper understanding of avian viruses both in captive and wild settings to implement appropriate biosecurity measures and aid in the conservation of vulnerable bird species.

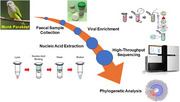

## 1 Introduction

Viral pathogens can present significant challenges, affecting everything from individual animals to whole ecosystems. Over the past few decades, significant efforts have been made to enhance our understanding of viromes in humans and animals. However, our knowledge of the diversity of viruses in birds remains limited. Most research on avian viruses has focused on zoonotic pathogens, such as avian influenza A virus (Lupiani and Reddy 2009), viruses causing economic losses in commercial poultry like Newcastle disease virus (NDV) and infectious bronchitis virus (Alexander 2000; Fabricant 1998), or those responsible for notable mortality in wild birds, such as Wellfleet Bay virus and beak and feather disease virus (BFDV) (Allison et al. 2015; Chang et al. 2020; Raidal, Sarker, and Peters 2015; Sarker, Edward, et al. 2014, Sarker, Ghorashi, et al. 2014). However, our knowledge of the viruses affecting many Australian birds, especially exotic species, is limited, hindering our ability to assess the potential risks and impacts these viruses may have on these species. The monk parakeet (*Myiopsitta monachus*) used in this study was obtained from a commercial trader without any information about their disease history. The monk parakeet (*M. monachus*) is recognised as a highly successful and widespread avian invader, attributed to the international trade of wild birds from their native South American habitats (Preston and Jones 2021; Cardador et al. 2017). They are considered an extreme pest risk if established in Australia due to their high climate match, generalist diet, potential to inflict significant damage to agricultural crops and documented history of successful establishment in numerous countries (https://www.daf.qld.gov.au). In addition, they can harbour zoonotic pathogens such as *Chlamydia psittaci*, avian influenza virus, NDV and enteropathogenic *Escherichia coli* (López et al. 2023). Monk parakeets also provide nesting opportunities for other non‐native species (Preston, Pruett‐Jones, and Eberhard 2021) and may serve as vectors for disease transmission to both native and invasive species, underscoring the importance of studying the virome in this invasive species.

Virome studies in various psittacine birds have revealed a plethora of viruses spanning the families *Adenoviridae*, *Circoviridae*, *Parvoviridae* and *Picornaviridae* (Athukorala et al. 2021; Sarker 2021a; Sarker and Phalen 2023; Sutherland and Sarker 2023). Psittacine beak and feather disease (PBFD) is particularly noteworthy as a chronic, often fatal viral infection that poses a significant threat to both wild and captive psittacine species worldwide (Bassami et al. 2001; Ritchie, Anderson, and Lambert 2003; Sarker, Ghorashi, et al. 2014). The causative agent, BFDV (species; *Circovirus parrot*), is a compact circular, ambisense single‐stranded DNA (ssDNA) virus of the genus *Circovirus* within the family *Circoviridae* (Niagro et al. 1998; Ritchie et al. 1990). Its rapid global spread is attributed to its high environmental persistence and ability to switch hosts (Sarker et al. 2015; Sarker, Ghorashi et al. 2014), posing significant concerns for agriculturists and conservationists alike. Continued research and surveillance on PBFD in invasive psittacine birds are imperative, particularly given the evident link between the exotic bird trade and the spread of novel BFDV strains to native bird populations.

In Australian psittacine birds, novel chaphamaparvoviruses (ChPVs) have been identified in asymptomatic rainbow lorikeets (Chang et al. 2020) and *Neophema* parrots (Klukowski et al. 2024; Sarker 2021b). Despite these findings, there is limited information on the presence and impact of parvoviruses in other non‐native psittacine species in Australia. In addition, a potential evolutionary relationship among the emerging psittacine adenovirus‐F, which is circulating in the critically endangered orange‐bellied parrot of Australia, and human adenovirus 2, and human mastadenovirus C highlighted the unexpected presence and genetic link of avian adenoviruses with human adenoviruses, suggesting a complex and intertwined virome, underscoring the need for further metagenomic detection and characterisation of adenoviruses in Australian psittacine and non‐psittacine birds (Sarker 2021). By expanding our understanding of the adenovirus diversity in these birds, we can gain insights into the potential for cross‐species transmission and the emergence of new viral strains that could impact both avian and human health.

Advancements in metagenomic sequencing techniques have revolutionised the exploration of viral communities inhabiting both animal and human hosts (Shi et al. 2016; Vibin et al. 2018; Vibin et al. 2020). Given the potential for conservation efforts of different parrot species in Australia, it is very much essential to understand the parrot viromes. Therefore, this study aimed to employ metagenomics to detect and characterise both known and unknown viruses that may be present in invasive parrot species, thereby enhancing our understanding of viruses that could pose risks to native species.

## 2 Materials and Methods

### 2.1 Sampling and Ethical Considerations

Fresh faecal samples were collected from four Monk parakeets at a pet shop in Melbourne, Australia. These samples were obtained from the cage floor during routine animal care and stored at −80°C in cryovials with RNA later until further processing. The sample collection process did not involve any handling or manipulation of the birds. The Animal Ethics Committee at La Trobe University was informed that the findings from this non‐invasive material (which did not involve direct contact with the birds) were intended for publication, and a formal waiver of ethics approval was granted. The samples were then subjected to molecular diagnostic tests at La Trobe University in Melbourne.

### 2.2 Virus Enrichment and Virus Nucleic Acid Extraction

After removing potential impurities such as host cells, bacteria, food particles and free nucleic acids from the faecal samples, the virus particles were then enriched using the methods described by Vibin et al. (2018), with slight modifications. Briefly, the faecal material was aseptically resuspended and vigorously homogenised in sterile phosphate‐buffered saline (PBS) at a 1:10 ratio. The suspension was then centrifuged at 17,000 × *g* for 3 min at room temperature. The resulting supernatant was filtered using a 0.80 µm syringe filter, and the filtrate was then processed downstream. Subsequently, the samples were ultracentrifuged at 178,000 × *g* for 1 h (30 psi for 1 h) at 4°C using the Hitachi Ultracentrifuge CP100NX. After centrifugation, the supernatant was discarded, and the pellet was resuspended in 130 µL of sterile PBS. The filtrates were then treated with nucleases by adding 2 µL of benzonase nuclease (25–29 U/µL, purity > 90%; Millipore) and 1 µL of micrococcal nuclease (2,000,000 gel units/mL; New England Biolabs), and incubated at 37°C for 2 h. The nuclease reaction was halted by adding 3 µL of 500 mM ethylenediaminetetraacetic acid (EDTA). Viral nucleic acids were then extracted using the QIAamp Viral RNA Mini Kit (Qiagen, Valencia, CA, USA) without the addition of any carrier RNA, allowing for the simultaneous extraction of both viral DNA and RNA. The quantity and quality of the isolated nucleic acids were assessed using a Nanodrop and an Agilent TapeStation (Agilent Technologies, Mulgrave, VIC, Australia) by the Genomic Platform at La Trobe University.

### 2.3 Next‐Generation Sequencing

Before library construction, the extracted nucleic acids were subjected to cDNA synthesis, and amplification was conducted using the Whole Transcriptome Amplification Kit (WTA2; Sigma‐Aldrich, Darmstadt, Germany) following the manufacturer's instructions. The amplified polymerase chain reaction (PCR) products were subsequently purified using the Wizard SV Gel and PCR Clean‐Up kit (Promega, Madison, WI, USA). The quantity and quality of the purified products were assessed using a Qubit dsDNA high‐sensitivity assay kit with Qubit Fluorometer v4.0 (Thermo Fisher Scientific, Waltham, MA, USA). Library construction was then carried out using the Illumina DNA Prep kit (Illumina, San Diego, CA, USA) according to the kit instructions, commencing with 250 ng of DNA as measured by a Qubit Fluorometer v4.0 (Thermo Fisher Scientific, USA). The quality and quantity of the prepared library were evaluated by the Australian Genome Research Facility in Melbourne, Australia. Subsequently, cluster generation and sequencing of the library were performed with 150 bp paired‐end reads using Illumina NovaSeq SP chemistry, following the manufacturer's instructions.

### 2.4 Bioinformatic Analyses

The resulting raw sequencing reads were analysed using an established pipeline (Athukorala et al. 2021; Sarker et al. 2017; Sarker et al. 2019; Sutherland et al. 2019) with Geneious Prime (version 2022.1.1, Biomatters, New Zealand). Briefly, the quality of all raw reads was assessed, and pre‐processing steps were taken to remove ambiguous base calls and poor‐quality reads, as well as to trim Illumina adapter sequences. The trimmed sequence reads were then mapped against the chicken genome *Gallus* (GenBank accession number NC_006088.5) to remove likely host DNA contamination. In addition, reads were further mapped to the *E. coli* bacterial genomic sequence (GenBank accession no. U00096) to remove possible bacterial contamination. The cleaned and unmapped reads were used as input data for de novo assembly using the SPAdes assembler (version 3.10.1) (Bankevich et al. 2012) with the ‘careful’ parameter in the LIMS‐HPC system (a High‐Performance Computer specialised for genomics research at La Trobe University). The resulting contigs were compared against the non‐redundant nucleotide and protein databases on GenBank using BLASTN and BLASTX (Benson et al. 2013), respectively, with an *E* value threshold of 1 × 10^−5^ to remove potential false positives. Contigs with significant BLAST hits corresponding to bacteria, eukaryotes or fungi were filtered out to exclude non‐viral reads. Virus contigs of interest greater than 300 nucleotides (nt) were imported into Geneious Prime (version 2022.1.1) for further functional analysis. The detected viruses were annotated using Geneious Prime (version 2022.1.1), where genus‐specific published viruses were used as a reference guideline.

### 2.5 Comparative Genomics and Phylogenetic Analyses

Genomic features of the newly sequenced viral genomes were analysed using Geneious Prime (version 2022.1.1). Sequence similarity percentages between representative viruses were determined using the tools available in Geneious Prime (version 2022.1.1). Amino acid sequences of protein‐coding genes and nucleotide sequences of selected partial genes were aligned using the MAFTT L‐INS‐I algorithm implemented in Geneious Prime (version 7.388; Katoh and Standley 2013). To identify the best‐fit model for constructing phylogenetic analyses, a model test was conducted in jModelTest 2 using default parameters (Darriba et al. 2012), favouring a general‐time‐reversible model with gamma distribution rate variation and a proportion of invariable sites (GTR + G + I). Phylogenetic analyses for nucleotide and protein sequences were performed using the GTR and WAG substitution models, respectively, with 1000 bootstrap support in the T‐REX web server (Boc, Diallo, and Makarenkov 2012). Maximum‐likelihood phylogenies of selected genomes in the present study were inferred using PhyML (version 3.1) (Guindon et al. 2005). Finally, the consensus tree was visualised and edited using FigTree V1.4.4 software. The tree was rooted at the midpoint and the branches were proportionally transformed.

## 3 Results

### 3.1 Detection of a Novel ChPV sp

The assembled genome of ChPV sp. is a linear ssDNA molecule consisting of 4309 nucleotides. Its basic organisation is consistent with other members of the *Parvoviridae* family. The genomic structure of ChPV is akin to that of other parvoviruses, featuring two primary open reading frames (ORFs) that encode a replication initiator protein (NS1) and a viral capsid protein (VP1). Comparative analysis using BLASTX and BLASTP identified significant protein sequence similarities (*E* value ≤ 10^−4^) for each of the four ORFs. The 5′ ORF1 spans 318 nucleotides, with its putative amino acid sequence showing 100% similarity to a hypothetical protein of ChPV sp. (GenBank accession no. WGD01533.1). The non‐structural protein 1 (NS1) ORF of ChPV, 1989 nucleotides long, exhibited the highest amino acid identity (99.38%) with ChPV sp. (GenBank accession no. WGD01535.1), followed by Ara ararauna ChPV (54.23%, GenBank accession no. QTE04010.1) and avian ChPV (54.24%, GenBank accession no. QKX49056.1). The NS1 gene of ChPV, like those in other parvoviruses, consists of 662 amino acids and encodes a helicase. This helicase includes the conserved ATP‐ or GTP‐binding Walker A loop (GPxNTGKT/S; _324_ **GP**S**NTGKS**_331_), Walker B (xxxWEE; _363_IGI**WEE**_368_) Walker B’ (KQxxEGxxxxxPxK; _380_ **KQ**VL**EG**MQTAI**P**V**K**_393_) and Walker C (PxxxTxN; _404_ **P**III**T**T**N**_410_) aa motifs. In addition, the NS1 protein features two conserved replication initiator (endonuclease) motifs: xxHuHxxxx (VF_113_ **H**V**H**_110_AMFQ) and YxxK (_166_ **Y**MM**K**_169_). In these sequences, bold letters indicate conserved amino acids, and ‘*u*’ represents a hydrophobic residue.

The major 3′ ORF of the ChPV genome, 1638 nucleotides in length, encodes a protein analogous to the VP1 capsid protein found in the *Parvoviridae* family. At the amino acid level, the ChPV VP1 protein is most similar to that of ChPV sp. (97.80% similarity, GenBank accession no. WGD01536.1), followed by psittaciform chaphamaparvovirus 3 (PsChPV‐3; 48.55% similarity, GenBank accession no. UNS41167.1), and *Psittacara leucophthalmus chapparvovirus* (48.80% similarity, GenBank accession no. YP_010798364.1). In addition, the ChPV genome contains a 540 nucleotide long ORF, which is homologous to the NP protein of turkey parvovirus 2 (60.89% similarity, GenBank accession no. YP_010796328.1), as well as to Galliform ChPV 7 (60.44% similarity, GenBank accession no. UOH27038.1) and psittaciform chaphamaparvovirus 5 (PsChPV‐5; 59.67% similarity, GenBank accession no. WOX03044.1).

**FIGURE 1:**
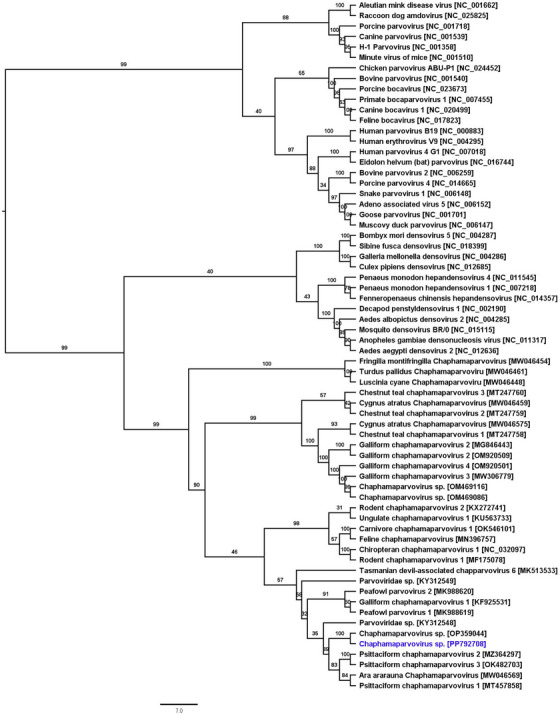
Phylogenetic tree depicting the possible evolutionary relationship between Chaphamaparvovirus (ChPV) and other selected parvoviruses. The ML tree was constructed using amino acid sequences of the complete NS1 gene. The numbers on the left show bootstrap values as percentages, and the labels at the branch tips refer to the original parvoviruses’ names, followed by their GenBank accession numbers in parentheses. The chaphamaparvovirus sp. sequenced in this study is highlighted in blue.

A phylogenetic analysis of parvoviral NS1 sequences clearly indicates that the newly sequenced *Chaphamaparvovirus* sp. belongs to the genus *Chaphamaparvovirus*. The maximum likelihood (ML) tree showed that the sequenced ChPV formed a cluster within the ChPV‐specific clade, along with other parvoviruses such as psittaciform chaphamaparvovirus 1, 2, and 3 (PsChPV‐1, PsChPV‐2 and PsChPV‐3), and Ara ararauna ChPV (see Figure 1). Using the same set of NS1 protein sequences, it was determined that the maximum interlineage sequence identity between the novel ChPV sp. and other psittaciform ChPVs ranged from 52.54% to 53.28%.

### 3.2 Evidence of Psittacine Adenovirus 5

In this study, a partial genome of the psittacine adenovirus 5 was sequenced from a monk parakeet. The 1059‐nucleotide‐long ORF1 exhibited 100% protein similarity to the penton protein of psittacine adenovirus 5 according to a BLAST search of the putative amino acid sequence (GenBank accession no. YP_010798276.1). It also showed high similarity to *Siadenovirus* sp. (99.53% protein similarity, GenBank accession no. QGZ10474.1), followed by psittacine adenovirus 6 (96.88% protein similarity, GenBank accession no. QIJ58766.1). Analysis of the 672‐nucleotide‐long ORF2 of psittacine adenovirus 5 revealed the closest match to the hexon protein of psittacine adenovirus 5 (99.81% protein similarity, GenBank accession no. AYJ76832.1), followed by budgerigar adenovirus 1 (99.78% protein similarity, GenBank accession no. QIG37590.1).

**FIGURE 2:**
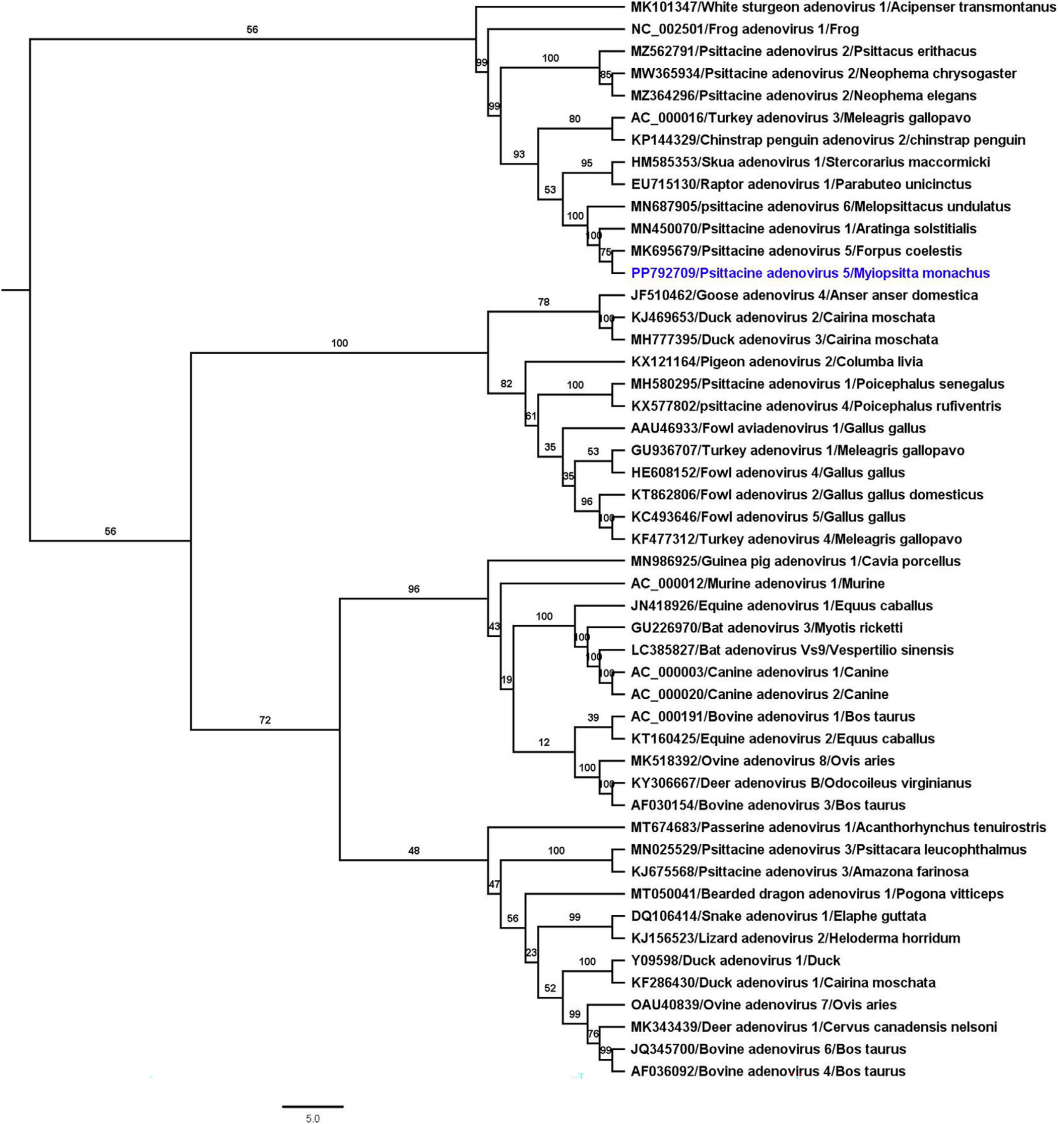
ML phylogenetic tree illustrating the possible evolutionary relationship of the psittacine adenovirus 5 with other selected adenoviruses. The phylogenetic tree was generated using a MAFFT alignment L‐INS‐I of nucleotide sequences of penton coding genes. The numbers on the left indicate bootstrap values as percentages and the labels at the branch tips refer to GenBank accession numbers followed by the names and original host species of the adenoviruses. The psittacine adenovirus 5 detected in this study is highlighted in blue.

Phylogenetic analysis based on penton coding gene sequences from selected adenoviruses placed psittacine adenovirus 5 within the Siadenovirus genus (Figure 2). The ML tree indicated that this virus formed a distinct clade with PsSiAdV‐1, PsSiAdV‐5 and PsSiAdV‐6 (GenBank accession nos. MN450070, MK695679, MN687905, respectively), with sequences recovered from faecal samples of a live monk parakeet from a pet shop showing 100% bootstrap support (Figure 2). Given the high sequence identities (>99%) and the phylogenetic placement among different PsSiAdV strains, it can be hypothesised that the psittacine adenovirus 5 detected in this study likely shares a common evolutionary origin.

### 3.3 Evidence of BFDV

**FIGURE 3:**
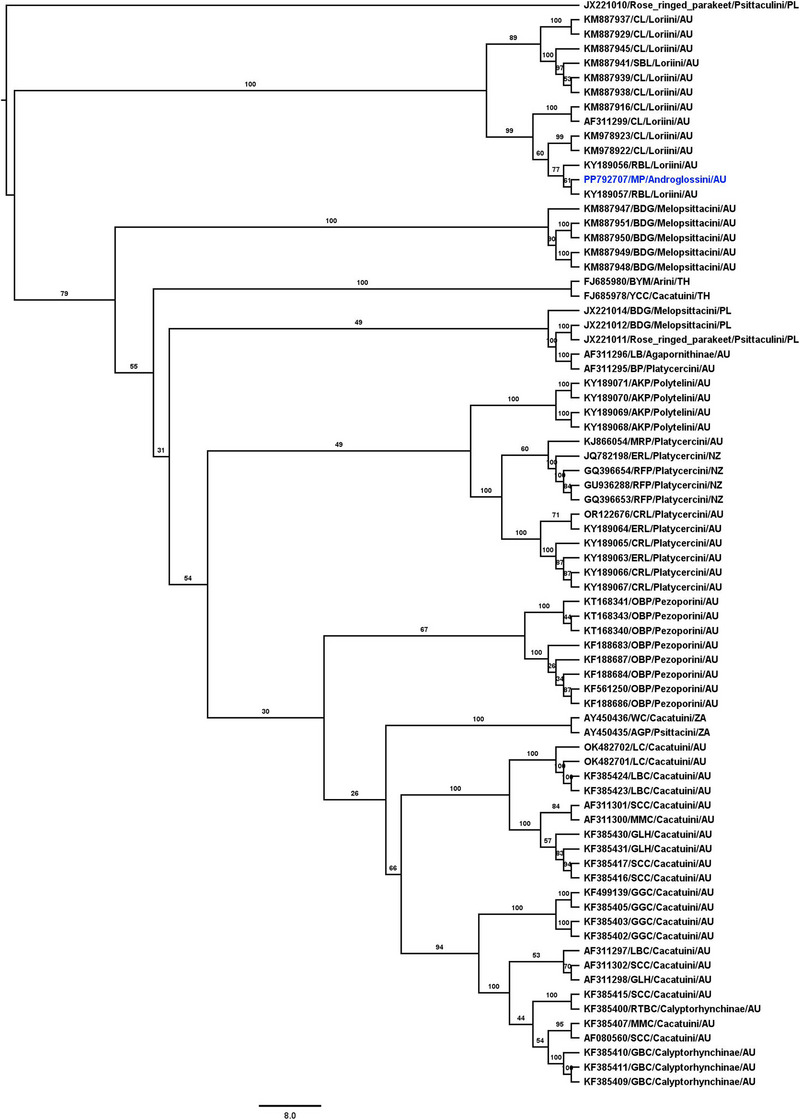
Phylogenetic tree showing the potential evolutionary relationships of the BFDV genomes detected in a monk parrot with other selected BFDV genomes. The BFDV detected in this study is highlighted in blue. AGP = African grey parrot; AKP = Australian king parrot; BDG = Budgeriger; BP = Bonnet parrot; CL = coconut lorikeet; CRL = crimson rosella; ERL = eastern rosella; GBC = glossy black cockatoo; GGC = gang gang cockatoo; GLH = galah; LB = love bird; LBC = long lilled corella; MMC = major Mitchell cockatoo; MP = monk parakeet; MRP = Mallee ring necked parrot; OBP = orange‐bellied parrot; RBL = rainbow lorikeet; RTBC = red‐tailed black cockatoo; SCC = sulphur‐crested cockatoo; WC = western corella.

The complete genome of BFDV identified in this study is 2141 nucleotides long (GenBank accession no. PP792707). This BFDV genome contains two bidirectional ORFs that encode the putative Rep and Cap proteins. Both the Cap and Rep proteins showed the highest sequence similarity to BFDV found in rose‐ringed parakeets (*Psittacula krameri*) from Australia, with 100% protein similarity (GenBank accession no. WOX03052.1). Phylogenetic analysis revealed that the BFDV genome sequenced in this study clusters with BFDV sequences from rainbow lorikeet and coconut lorikeet in Australia (Figure 3).

## 4 Discussion

Recent advances in molecular techniques have significantly broadened our understanding of avian viruses, identifying numerous novel avian virus species. However, the viruses currently recognised likely represent only a fraction of the wide diversity of avian viruses. This indicates that there is a considerable amount of viral diversity within avian populations yet to be unveiled. In this study, we have identified three viruses—ChPV, BFDV and psittacine adenovirus 5 in faecal samples from Monk parakeets (*M. monachus*) in Melbourne, Australia. Metagenomic sequencing has facilitated the detection of several novel parvoviruses, some of which have been found in avian species (Kim et al. 2020; Liu et al. 2020; Matos et al. 2022; Sarker 2021; Sarker et al. 2022). The ChPV sp. discovered in this investigation has been categorised within the recently established *Chaphamaparvovirus* genus of the *Hamaparvovirinae* subfamily. This subfamily includes parvoviruses that infect a range of hosts, spanning from vertebrates to invertebrates, and share more than 30% amino acid sequence identity in the NS1 protein (Pénzes et al. 2020). The parvovirus found in the faecal samples of the Monk parakeet shows the closest phylogenetic resemblance to PsChPV‐3, sharing a genome identity of 66.89% and an amino acid sequence identity of 53.73% for the NS1 protein. The clinical implications and pathogenic mechanisms of ChPV in monk parakeets are still uncertain, highlighting the necessity for further investigation into this new avian virus in both wild and captive bird populations. Such research is crucial for enhancing our understanding of its evolution and potential pathogenic impacts.

The resulting ML tree of adenoviruses (Figure 2) showed that the psittacine adenovirus 5, detected in the monk parakeet formed a monophyletic clade with PsAdV‐1, PsAdV‐5 and PsAdV‐6. The naturalisation of this invasive species in Australia poses a significant threat to native parrot species and predatory birds, which may harbour the potential risk of introducing psittacine adenovirus 5 into the wild population, adversely affecting their survival and reproduction. Therefore, regular monitoring of the health of captive monk parakeets is crucial to prevent the spread of viral diseases and to protect endangered wild bird species. Nonetheless, determining the precise biological consequences of the psittacine adenovirus 5 from this study presents challenges, indicating the need for further research to understand the virus’ impact fully.

BFDV is recognised as a significant wildlife pathogen that causes PBFD, affecting a wide variety of Psittaciformes (Raidal, Sarker, and Peters 2015; Todd 2000). BFDV, believed to have originated and co‐evolved among Australian parrots, has rapidly disseminated worldwide, a spread significantly fuelled by the international trade of exotic Psittaciformes, both legal and illegal (Amery‐Gale et al. 2017; Fogell, Martin, and Groombridge 2016; Fogell et al. 2018). Besides, the virus’ ability to persist in the environment and its capacity for flexible host switching have further facilitated its worldwide spread, posing a significant threat to global parrot conservation programs (Fogell, Martin, and Groombridge 2016; Raidal and Peters 2018). Previous studies have shown that individual Australian birds can be infected with multiple variants of BFDV. In some cases, about 30 different variants of BFDV have been identified across nine individual birds, with one particular bird found to be carrying up to seven genetic variants of the BFDV (Sarker et al. 2014). Phylogenetic analysis based on the selected complete genome sequences of BFDV revealed that the BFDV genomes sequenced from monk parakeets clustered with the BFDV sequences from rainbow lorikeet and coconut lorikeet. Instead, these genomes shared closer genetic relationships with BFDV genotypes found in rose‐ringed parakeets and budgerigars from Poland (Figure 3), suggesting that the BFDV genomes obtained in our study are likely to be recombinant sequences brought about by the import and trade of exotic parrots. This finding underscores the impact of wildlife trade on viral evolution and the potential risks associated with the introduction of novel pathogens through such practices. Furthermore, the presented phylogenetic tree is supported by a previous study (Das et al. 2016), which demonstrated that BFDV sequences from lorikeet species formed distinct tribe‐specific clusters, indicating independent evolutionary pathways. Conversely, BFDV sequences from other host species displayed characteristics of host generalisation and frequent genetic mixing across populations through inter‐lineage recombination, suggesting a complex pattern of BFDV evolution driven by host interactions (Das et al. 2016). These findings further highlight the adaptability of BFDV, facilitated by its ability to transmit horizontally and survive in the environment for extended periods, raising concerns about its impact on diverse avian populations both within Australia and globally (Raidal and Cross 1994; Sarker et al. 2014).

Wild avian hosts play a crucial role as natural reservoirs and potential vectors for various infectious disease viruses, with emerging viruses posing significant risks to human, animal and environmental health (Chang et al. 2020; Sarker 2021; Sutherland et al. 2019). Australia, home to over 370 parrot species, faces significant conservation challenges, with 85 species listed as critically endangered or vulnerable and 19 on the brink of extinction (IUCN 2022). BFDV is considered a major biosecurity threat due to the potential risks pretended by parrot conservation programs (Australian Government 2022). Therefore, there is still great urgency in the ongoing identification of novel pathogenic variants and potential pathogens in free‐ranging birds. BFDV, for instance, may persist in environments like nest hollows, contributing to the spread of the virus and facilitating the emergence of new genotypes (Sarker et al. 2014). Our findings underscore the critical need for ongoing surveillance and molecular epidemiology research on viruses like ChPVs, adenoviruses, and BFDV, as the ecological impacts of these viruses could significantly impede recovery efforts for endangered Australian birds. The present study was limited by using a small number of birds at a specific time point. Further research is needed using sampling birds over time from both the wild and pet birds across various locations in Australia.

In conclusion, the virome of parrots emerges as a vast reservoir of viral diversity with significant implications for avian health. Ongoing research focusing on the composition, dynamics, and evolution of parrot viromes is indispensable. Such efforts not only aid in mitigating zoonotic risks but also enhance our understanding of avian viral ecology. In addition, such research plays a pivotal role in informing and bolstering conservation strategies aimed at protecting these diverse and fascinating avian species.

## Author Contributions

**Babu Kanti Nath**: conceptualisation, methodology, validation, formal analysis, data curation, writing–original draft preparation and writing–review and editing. **Suman Das Gupta**: validation and writing–review and editing. **Saranika Talukder**: resources and writing–review and editing. **Nasrin Sultana Tonu**: validation and writing–review and editing. **Shane R. Raidal**: resources and writing–review and editing. **Jade K. Forwood**: resources and writing–review and editing. **Subir Sarker**: conceptualisation, methodology, validation, formal analysis, investigation, resources, data curation and writing–review and editing.

## Consent

The authors have nothing to report

## Conflicts of Interest

The authors declare no conflicts of interest.

### Peer Review

The peer review history for this article is available at https://publons.com/publon/10.1002/vms3.70083.

## Data Availability

The GenBank accession numbers for the viral sequences are PP792707, PP792708 and PP792709. The SRA accession number is SRR26413810. The Bioproject number is PRJNA1028305, and the Biosample number is SAMN37819503.
